# Evaluation of the Tensor Fascia Lata Muscle Pedicle Bone Grafting Technique in the Management of Avascular Necrosis of the Femoral Head: An Observational Clinical Study

**DOI:** 10.7759/cureus.64902

**Published:** 2024-07-19

**Authors:** Navin Kumar Yadav, Pavan Pradhan, Madhur Anand Pratap Singh, Surendra Kumar, Ashok Yadav

**Affiliations:** 1 Department of Orthopaedics, Baba Raghav Das Medical College, Gorakhpur, IND

**Keywords:** anterolateral approach to the hip, ficat-arlet stage, tensor fascia lata (tfl) muscle pedicle iliac crest bone grafting, harris hip score, avascular necrosis femoral head

## Abstract

Background: Osteonecrosis of the hip is defined as necrosis of the bone tissue due to some form of vascular insult, subsequently leading to the collapse of the femoral head and secondary osteoarthritis, which leads to pain and impaired joint function. This disease is widely known to affect middle-aged groups; however, in the Indian population, even younger people are more commonly affected. The disease has a debilitating effect on the activities of daily living (ADL) and the productivity of individuals and has financial consequences. With the increased utilization of magnetic resonance imaging (MRI) in society, the disease is diagnosed in its early stages. Hip-preserving surgery like tensor fascia lata (TFL) muscle pedicle iliac bone grafting should be given a chance to preserve the native femoral head.

Methodology: At a tertiary care teaching hospital in Gorakhpur, India, an observational clinical study was carried out. This study comprised 40 patients, ages 18-50 years, with femoral head osteonecrosis (stages II and III of the Ficat-Arlet staging system), who came to our institute's orthopedic outpatient department. Patients were treated with multiple drillings, curettage, and cheilectomy of the femoral head, in addition to TFL muscle pedicle bone grafting. The Harris hip score (HHS) was utilized to assess the clinical results, and the radiological assessment focused on signs of revascularization.

Result: In our study, the most prevalent age group was 20-30 years (67.5%), with a male predominance (85%). Among our cohort of 40 patients, the HHS indicated excellent outcomes (90-100) in 14 cases (35%), good outcomes (80-89) in 19 cases (47.5%), fair outcomes (70-79) in six cases (15%), and poor outcomes (<70) in one case (2.5%), at the time of the final follow-up. The final follow-up period varied from one to 10 years.

Conclusion: TFL muscle pedicle bone grafting procedure provides excellent clinical and radiological outcomes, especially in young patients in whom femoral head-preserving surgery is preferred over total hip arthroplasty. This procedure is effective in both early and advanced stages of femoral head osteonecrosis, provided there are no arthritic changes. It reduces symptoms and improves functional outcomes.

## Introduction

Osteonecrosis of the hip, also referred to as aseptic necrosis or avascular necrosis (AVN) of the hip, is a disease where bone tissue dies due to the disruption of the blood supply of the femoral head. Spontaneous remission of osteonecrosis of the hip is uncommon [1]. There are several underlying conditions that can cause femoral head osteonecrosis, including hip joint trauma or surgery, corticosteroid use, irregular blood pressure, smoking, excessive alcohol consumption, endotoxin poisoning, autoimmune disorders, hyperlipidemia, COVID-19, using contraceptives, pregnancy, and blood hypercoagulability [2]. The common etiological factors are chronic alcoholism (20.1% cases), followed by idiopathic (21.3% cases), and almost 37.3% of cases possess a history of prolonged steroid use [3].

Early adulthood and middle age groups have a markedly increased risk of osteonecrosis in comparison to children because AVN in children always heals. In India, the ratio of men to women is 5:1, and the average age at which femoral head necrosis usually appears is 32±2.5 years [3]. Studies on the prevalence of femoral head osteonecrosis in the Indian population are limited [3]. On the other hand, in the United States, about 10,000-20,000 new patients with AVN of the femoral head are diagnosed annually. The condition mostly affects people between the ages of 20 and 50 years, and 5-12% of AVN cases ultimately require total hip arthroplasty (THA) [4].

Femoral head osteonecrosis significantly impairs individuals during their prime working years, and it is a prevalent factor driving hip arthroplasty among the young. The disease may involve both hips in 40-70% of patients [5]. A good prognosis for the condition often depends on timely clinical and radiological diagnosis, which is typically challenging but could prevent further femoral head collapse [6]. In clinical practice, the most frequently applied classification system of femoral head osteonecrosis is the Ficat-Arlet, which relies on standard X-rays [7]. The early diagnosis of patients in pre-arthritic stages has been made possible by the increasing availability of magnetic resonance imaging (MRI) [8].

The clinical manifestation is almost atypical, mostly consisting of groin pain radiating to the knee. Particularly in internal forced rotation, a restricted range of motion (ROM) in the hip is observed. Considering that the other side may also be involved, a patient's history is essential for generating suspicions about the illness and for further investigation [9]. While the general consensus for the treatment of femoral head osteonecrosis remains elusive, optimal outcomes hinge on early intervention, ideally before femoral head collapse occurs.

Early diagnosis is essential since it mostly affects people in early adulthood and middle age. If early treatment is not received, femoral head collapse will occur in up to 80% of individuals [10]. Head preservation procedures are recommended in the initial stages of the AVN because they reduce intraosseous pressure and enhance the blood supply of the femoral head [11].

This study aims to assess the effectiveness of the tensor fascia lata (TFL) muscle pedicle iliac bone grafting procedure in the management of femoral head AVN. Muscle pedicle bone grafting has become a popular treatment option for this illness, and it has been used in a number of institutions recently with varying degrees of success. The effectiveness of the procedure in re-establishing the affected femoral head blood supply is a measurable parameter, and it is still up for discussion.

## Materials and methods

In a tertiary care teaching hospital in Gorakhpur, India, this observational study was carried out after receiving approval from the Institutional Ethics Committee of Baba Raghav Das Medical College and Nehru Hospital (approval number: EC/NEW/INST/2020/1275). This study comprised 40 patients (44 hips), who had osteonecrosis of the femoral head (up to grades II and III of the Ficat-Arlet staging), with an age range of 18-50 years (average age of 28.4 years), and underwent treatment using the TFL muscle pedicle iliac bone grafting procedure. Additionally, the treatment regimen included the implementation of various surgical procedures, such as multiple drillings, curettage, and cheilectomy of the femoral head. The final follow-up duration varied from one to 10 years (average final duration 4.3 years). A comprehensive historical account was acquired through the utilization of a study proforma, with a specific focus on the underlying mechanisms involved in the development of the femoral head AVN. The assessment of additional correlated symptoms relies on the analysis of the medical history, findings of the physical examination, and relevant medical records of the patients. Written consent for surgery was obtained from all patients who were enrolled in this study. The benefits and drawbacks of the procedure were thoroughly explained to both patients and their companions. The concept of the risk-benefit ratio was also elucidated to them. An MRI of both hips and a standard radiograph of the pelvis with both hips were used to confirm the AVN stage prior to surgery. After the completion of the preoperative workup and providing written and informed consent, the patients were taken for the operative procedure. The outcomes were assessed using radiological evidence of revascularization, and the Harris hip score (HHS) [12] was utilized for clinical evaluation.

Study tools

The study tools used were HHS and the Ficat-Arlet staging system. The parameters of HHS were as follows: (1) pain, (2) limp, (3) distance, (4) support, (5) sitting, (6) enter public transportation, (7) stairs, (8) put on shoes and socks, (9) absence of deformity, and (10) ROM. The grading of HHS was as follows: <70 points: poor; 70-79 points: fair; 80-89 points: good; and 90-100 points: excellent. The preoperative radiological progression was evaluated using the Ficat-Arlet staging system [7], in the following manner: stage I: normal radiographs; stage II: density change in the femoral head, sclerosis or cystic changes, normal joint line, and normal head contour; stage III: femoral head flattening (crescent sign), loss of sphericity, and collapse; and stage IV: joint space narrowing with arthritic changes in the acetabulum.

Inclusion criteria

This study included 40 cases (44 hips) with AVN of the femoral head, patients of both sexes, aged between 18 and 50 years, and patients with osteonecrosis of the femoral head Ficat-Arlet stages II and III.

Exclusion criteria

The study excluded individuals with chronic hepatorenal disorders, hips with implants, hips that had undergone previous surgery, the presence of an active infection, and Ficat-Arlet stage IV.

Surgical procedure

**Figure 1:**
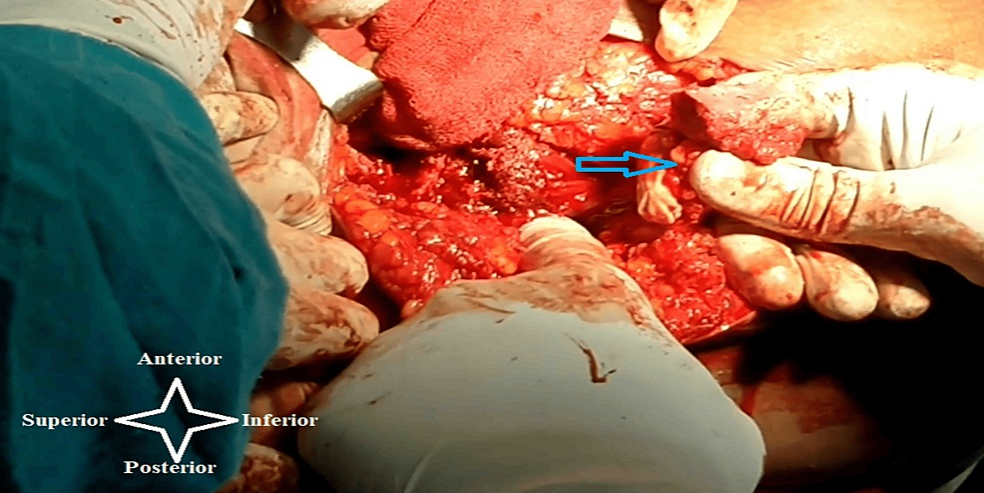
Harvesting of the iliac crest bone graft with the attached tensor fascia lata muscle (blue arrow) The tensor fascia lata muscle primarily receives its blood supply from the ascending branch of the lateral circumflex femoral artery, although it is also supplied by a branch of the superior gluteal artery

An anterolateral approach to the hip was used to enhance the exposure of the femoral head and neck, where the patient was lying supine on a traction table. This approach involved minimum exposure of the anterolateral side of the hip joint in order to preserve the posterior retinacular arteries and direct the operating surgeon to the anterolateral quadrant of the femoral head, which is the portion of the femoral head that is most frequently affected. To reveal the necrotic area that was removed, a 15×15 mm window was made close to the head-neck junction. Numerous drill holes were created through this window in order to improve revascularization. Subsequently, the skin incision was enlarged to the middle one-third region of the iliac crest, and a 20×20 mm bone containing the TFL muscle pedicle was cautiously separated and rotated downward, making sure the vascular pedicle had not been overstretched (Figure 1). The component of bone was secured with a 4 mm cannulated screw after being molded to fit inside the window. Capsule repair was done, and the wound was meticulously closed, layer by layer, with a suction drain in situ.

Postoperative management

Patients were permitted to sit up in bed 24 hours post-surgery. The first postoperative dressing and drain removal occurred within 48 hours. The operated hip was positioned with 30 degrees of flexion and neutral rotation. This position was recommended for about a week to relieve tension in the vascular pedicle. Static exercises for the thigh and gluteal muscles were initiated the next day after the surgery, when the pain subsided. On the 10th day following surgery, the sutures were taken out. ROM exercises could start after a fortnight. Up to the sixth postoperative week, toe touch-down non-weight bearing was permitted. After that, up to 12 weeks, crutches were allowed for gradual weight bearing. After 15-24 weeks, unsupported full-weight bearing was allowed.

Follow-up interval

A regular follow-up was conducted after six weeks, 12 weeks, 24 weeks, and one year, but in some patients, the final follow-up periods varied up to 10 years. A follow-up assessment of the patients was carried out using the HHS. X-rays were analyzed for signs of osteoarthritic changes. Bone structure improvement or no change was considered a favorable outcome, while the development of osteoarthritis was considered a poor outcome.

## Results

Forty patients (44 hips) with femoral head osteonecrosis (Ficat-Arlet stages II and III) were chosen in accordance with the selection criteria. Table 1 shows the age group distribution of the cases. The majority of the cases in this study were obtained from the age group of 20-30 years.

**Table 1:** Age group distribution of the cases

Age group (years)	No. of cases	%
Below 20	4	10
20-30	27	67.5
30-40	4	10
40-50	5	12.5

Table 2 shows the gender distribution of the cases. The majority of the cases in this study were obtained from the male gender.

**Table 2:** Gender distribution of the cases

Sex	No. of cases	%
Male	34	85
Female	6	15

Table 3 shows the side distribution of the cases. The majority of the cases in this study were obtained from the left side of the hip.

**Table 3:** Side distribution of the cases AVN: avascular necrosis

Side of AVN of the hip	No. of cases	%
Right	16	40
Left	20	50
Bilateral	4	10

Table 4 shows the distribution of potential risk factors of the cases. Seventeen patients were alcoholic, 16 patients had a history of steroid use, and seven patients had no known risk factors.

**Table 4:** The etiologies or potential risk factors for femoral head AVN AVN: avascular necrosis

Causes of AVN	No. of cases	%
Alcoholism	17	42.5
Corticosteroids	16	40
Idiopathic	7	17.5

Table 5 shows the distribution of the cases based on the Ficat-Arlet staging. The majority of the cases in this study were in stage III.

**Table 5:** Distribution of the cases according to the Ficat-Arlet staging

Ficat-Arlet staging	No. of cases	%
II	7	17.5
III	33	82.5

In our series of 40 patients, the HHS was excellent (90-100) in 14 (35%) cases, good (80-89) in 19 (47.5%) cases, fair (70-79) in six (15%) cases, and poor (<70) in one (2.5%) case at the final follow-up (Table 6).

**Table 6:** Evaluation of the Harris hip score in the study cases

Harris hip score	Result	No. of cases	%
90-100	Excellent	14	35
80-89	Good	19	47.5
70-79	Fair	6	15
<70	Poor	1	2.5

Radiographs

**Figure 2:**
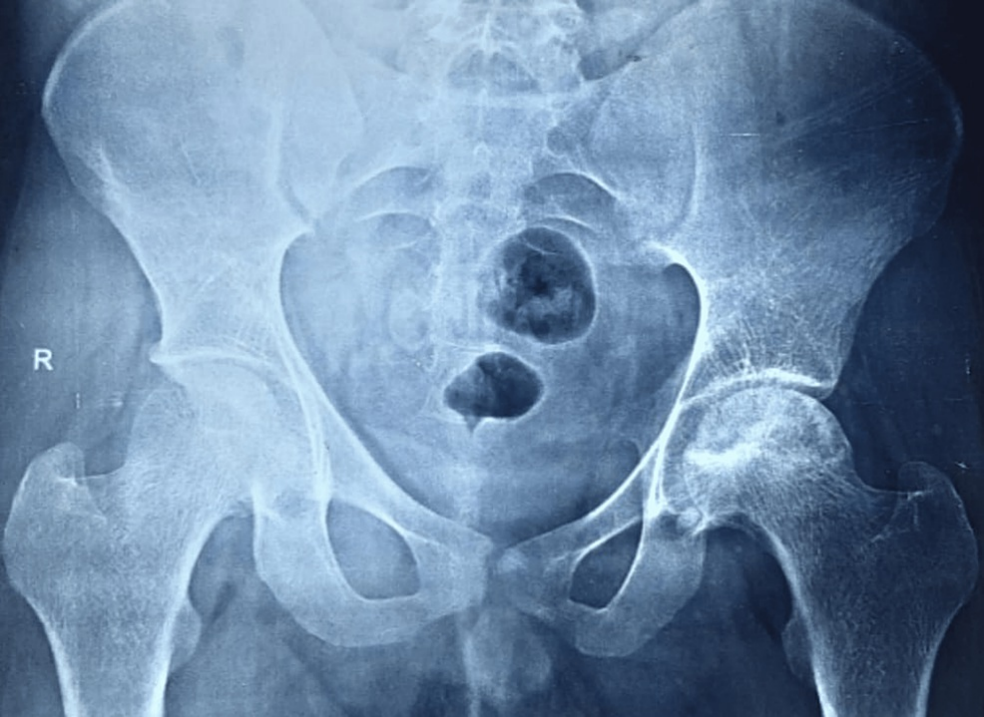
Preoperative radiograph of the pelvis with both hips (anteroposterior view), showing osteonecrosis of the left femoral head (Ficat-Arlet stage III)

**Figure 3:**
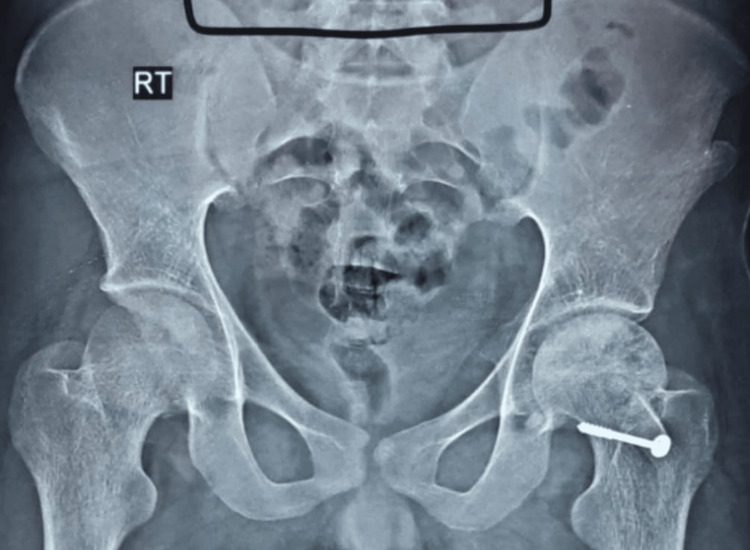
Postoperative radiograph at six months of follow-up

**Figure 4:**
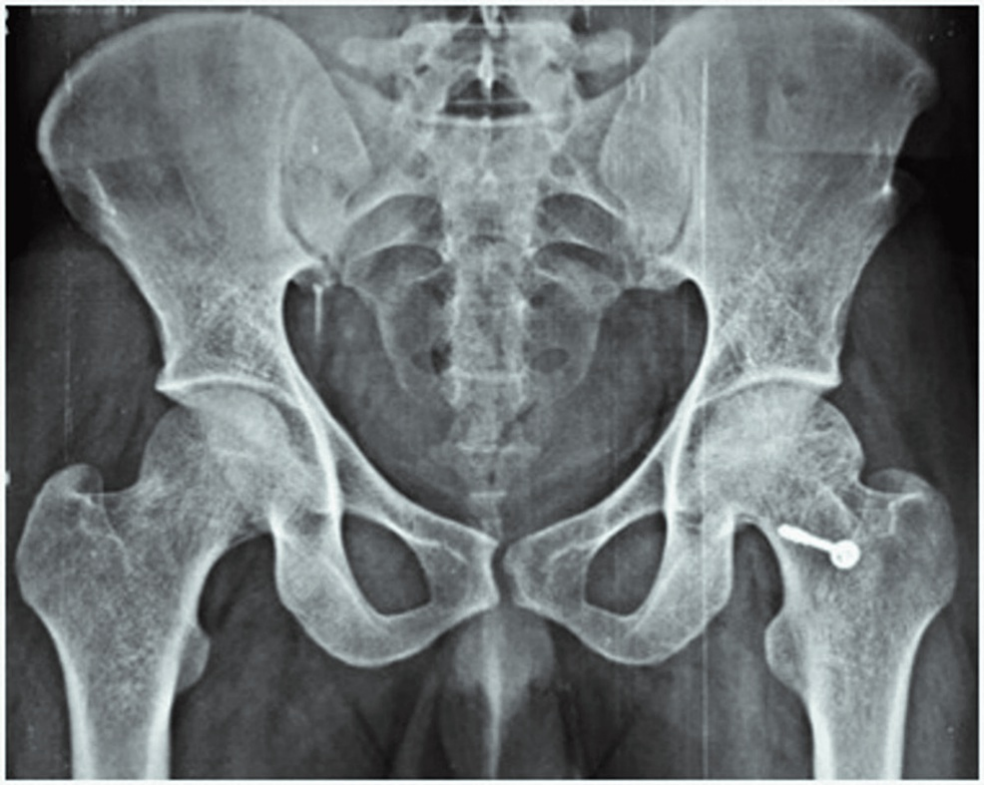
Postoperative radiograph at five years of follow-up

**Figure 5:**
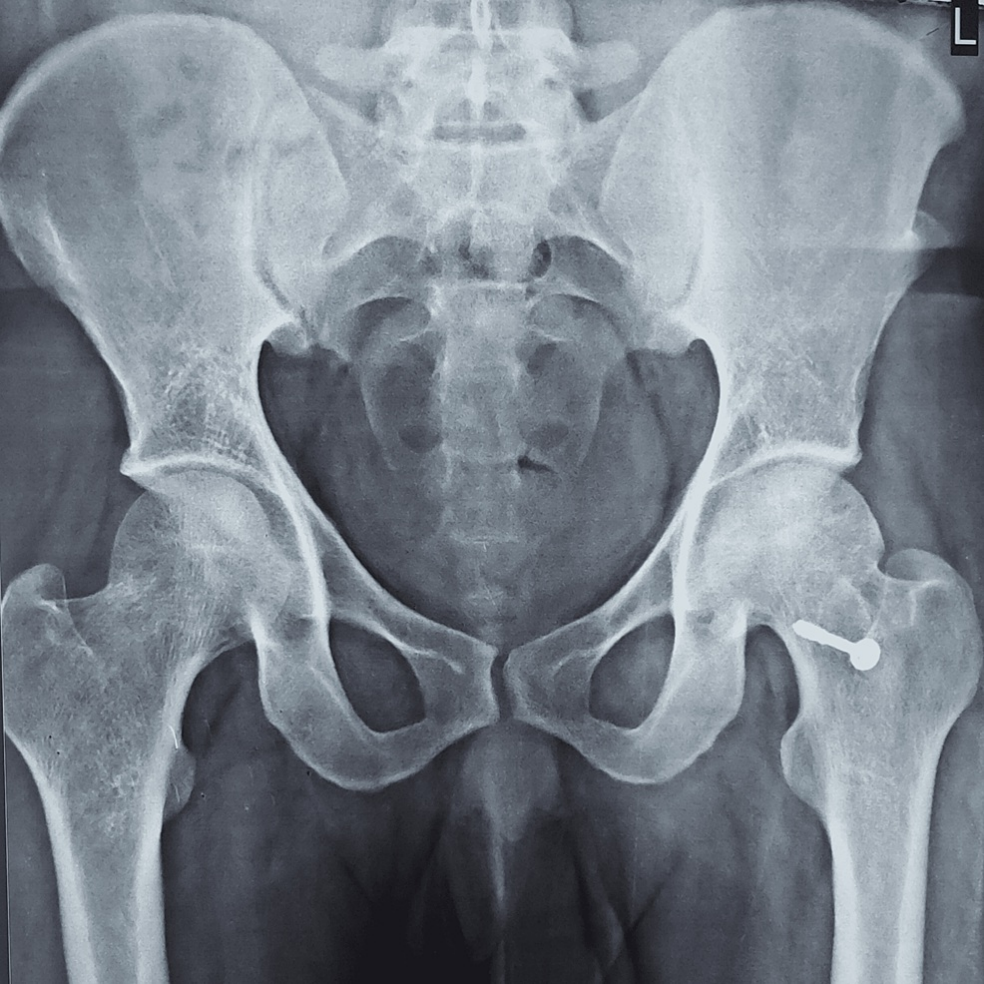
Postoperative radiograph at 10 years of final follow-up

Radiographic images of a patient, which include preoperative, postoperative, and final follow-up X-rays, are displayed in Figures 2-5. The patient who underwent surgery for femoral head osteonecrosis with TFL muscle pedicle iliac bone grafting demonstrated a notable improvement in radiographic evaluation.

## Discussion

In India, the TFL muscle pedicle bone grafting, an effective hip-preserving procedure for femoral head AVN, was first popularized by Baksi [13]. Hip-preserving surgical procedures are helpful for individuals who developed femoral head osteonecrosis before the age of 50 years, as these procedures can postpone THA by a decade or two. Many hip-preserving approaches have been suggested for femoral head AVN in the young population, but none of these have shown consistent and long-lasting outcomes [13].

Medical therapy for hip osteonecrosis is typically ineffective, although some studies have demonstrated the effectiveness of bisphosphonates in the early stages of osteonecrosis with a small area of involvement [14].

Core decompression is the cornerstone of treatment for femoral head osteonecrosis. However, according to certain studies, 37% of patients following core decompression experienced femoral head collapse and underwent THA [15]. The rationale could be that the subchondral cortical plate and the femoral head mechanical support structure could be destroyed by the bone defect brought on by core decompression, increasing the probability of femoral head iatrogenic collapse for advanced femoral head AVN [16].

In recent years, porous tantalum implants with core decompression have been used to treat femoral head osteonecrosis. But Floerkemeier et al. observed a 56% failure rate after implanting a tantalum rod in osteonecrosis of the femoral head. In addition, the surgery entails higher expenses and longer operation times [17].

Rotational osteotomies move the osteonecrotic part of the femoral head away from the primary weight-bearing area of the acetabulum. However, pseudoarthrosis and bony nonunion are the specific complications [18]. According to Langlais et al., these operations are technically challenging, may put vascularity at further risk, and have variable outcomes (only 47% favorable results) based on the size of the osteonecrotic part, types, and degree of required rotation [19].

Nonvascularized bone grafts (tibia, fibula autograft, or allograft) have been hardly used in recent years due to their high failure rate. Keizer et al. treated osteonecrosis of the femoral head using tibia autografts in 18 patients and fibula allografts in 62 patients. Of the 78 hips available for examination, 42 patients (54%) demonstrated clinical failure [20].

Vascularized fibular grafts have been used for the management of osteonecrosis, which has increased recently, with differing degrees of success. The process takes a long time, requires a high level of technical skill, and has a higher learning curve. Some studies have reported a 5-30% failure rate of this procedure [21,22].

Femoral head AVN is a progressive and multifactorial disorder that can eventually require a THA due to fragmentation, collapse of the femoral head, joint destruction, and degenerative changes. Young individuals have a significant incidence of femoral head osteonecrosis.

Due to the higher life expectancy of the patients and the maximum 10-year survival of implants, revision arthroplasty is almost always necessary in cases involving such young patients. Therefore, femoral head preservation and salvage procedures are required.

In numerous series, the typical age of a THA in AVN is about 38 years. The results are disappointing in the younger age group due to the higher rate of complications and the further need for revisions, mainly if carried out for osteonecrosis of the femoral head. While there is increasing proof of improved outcomes using modern elements and methods, treating hip osteonecrosis with a THA is still a significant problem. There is ongoing debate on the necessity of hip replacement at particular AVN stages in young patients [23].

Many authors have published descriptions of muscle pedicle bone grafts in the literature. Meyer described a pedicle graft based on the quadratus femoris. He concluded that, in the initial stages of osteonecrosis, the utilization of quadratus femoris muscle pedicle bone graft along with free cancellous graft yielded favorable outcomes, but in the more advanced stages, the results were unsatisfactory [24].

TFL muscle pedicle bone grafting offers significant benefits to hips in stages II and III. It utilizes a portion of the iliac crest, is simple to perform, and does not require any particular equipment or technique while also providing the benefits of enhanced blood supply, similar to a vascularized bone graft [25].

Baksi reported the results of using various types of muscle pedicle bone grafting procedures (quadratus femoris, gluteus medius, sartorius, or TFL) in various stages of femoral head AVN. According to the study, because of their good vascularity and corticocancellous nature, TFL muscle pedicle bone grafts in pre-collapse osteonecrosis provide a good strut effect to the subchondral region and prevent collapse with greater efficiency than those produced by other muscle pedicle bone graft techniques [13].

Increased vascularity and prevention of future head collapse can be achieved with a TFL muscle pedicle bone graft placed on the affected femoral head. For the ordinary orthopedic surgeon, the operation has a less steep learning curve and is technically less challenging, but it also has a higher reward rate.

In our series, every patient in stage II and one-third of patients in stage III had outstanding radiological and clinical outcomes. The majority of cases (67.5%) fell within the 20-30 age group, with an average age of 28.4 years. According to Langlais et al. [19] and Babis and Soucacos [23], the mean age was 27 years and 26 years, respectively, in their study.

The cause of AVN of the hip is complex and associated with various risk factors. In our study, alcohol consumption emerged as the leading cause In 17 cases (42.5%), followed by corticosteroid use in 16 cases (40%) and idiopathic origins in seven cases (17.5%).

The male gender dominated the sample, accounting for 85% of cases, while females constituted the remaining 15%. This gender distribution reflects a lower representation of female participants, possibly due to restricted patient availability at the hospital. Lakshminarayana et al. [26] also found similar observations in their study.

This study focused solely on the Ficat-Arlet staging system (stages II and III), with a prevalence rate of 17.5% for stage II and 82.5% for stage III. The average follow-up period was 4.3 years. The patient's age or gender did not appear to have any bearing on the outcome. Etemadifar et al. [27] also could not find any correlation between outcomes and sex.

This study investigated 40 cases of AVN affecting femoral heads. Among these cases, 16 cases (40%) were identified on the right hip and 20 cases (50%) on the left hip, while four cases (10%) showed bilateral hip involvement.

Among our cohort of 40 cases, the clinical outcomes were excellent in 14 cases (35%), good in 19 cases (47.5%), fair in six cases (15%), and poor in one case (2.5%), at the time of the final follow-up. The follow-up period varies from one to 10 years.

The radiological recovery of all stage II had been demonstrated by the fading of the sclerotic region and the retention of the head shape in homogeneous joint space. It was found that six hips in stage III had radiological evidence of the disease's progression during the follow-up period.

Thus, our study shows that the TLF muscle pedicle bone grafting technique can prevent head collapse, slow the advancement of the AVN of the hip, and minimize the need for a THA in young individuals. It also alleviates pain and enhances functional results. 

Limitations of this study

The study involved a small population from only a single center. Individuals have different follow-up periods, and the distribution of patients at each stage of the disease under evaluation is not consistent. So more patients and regular follow-up are needed to evaluate long-term functional and radiological outcomes.

## Conclusions

Our observations indicate that the TFL muscle pedicle bone grafting procedure provides excellent clinical and radiological outcomes, especially in young patients in whom femoral head-preserving surgery is preferred over THA. This procedure is effective in both early and advanced stages of femoral head osteonecrosis, provided there are no arthritic changes. In most patients, it reduces symptoms and improves functional outcomes. However, further longitudinal studies with larger sample sizes are necessary to draw definitive conclusions.
